# Chronic bilateral dacryoadenitis caused by SARS-CoV-2 infection: a case report

**DOI:** 10.1186/s13256-023-04175-7

**Published:** 2023-10-24

**Authors:** Bassem Awada

**Affiliations:** Division of Infectious Diseases, Department of Internal Medicine, Sultan Qaboos Comprehensive Cancer Care and Research Center, Al Khoud, PO Box 566, Muscat, Sultanate of Oman

**Keywords:** Case report, SARS-CoV-2, Dacryoadenitis, Lacrimal gland, Inflammation

## Abstract

**Background:**

Dacryoadenitis is inflammation of the lacrimal gland, mainly caused by viral infection. It can also be caused by bacterial pathogens and non-infectious processes such as auto-immune diseases and malignancy. Chronic dacryoadenitis is rarely linked to SARS-CoV-2 infection, with only five reports in the literature.

**Report:**

A 26-year-old Arab woman experienced chronic inflammatory dacryoadenitis after a mild SARS-CoV-2 infection, which was successfully treated with oral prednisone.

**Conclusions:**

Dacryoadenitis can occur due to inflammation caused by either SARS-CoV-2 exposure. The treatment typically involves the administration of steroids, with duration to be decided based on clinical response.

## Introduction

The emergence of the novel coronavirus (SARS-CoV-2) had significantly impacted the global health causing the most devastating pandemic in human history. The patients with SARS-CoV-2 infection presents with a range of mild upper respiratory symptoms to a severe life threatening viral pneumonia. Aside from the respiratory symptoms, SARS-CoV-2 is well-known to affect various organs, including the ocular system. Several observational studies reported that patients with SARS-CoV-2 infection may experience ocular symptoms, with rates ranging from 2 to 32% [1, 2]. The most common of these symptoms is conjunctivitis [1, 2]. To a lesser extent, other ocular manifestations, such as episcleritis, retinal vein thrombosis, cellulitis, and rarely dacryoadenitis, have also been documented [1, 2]. Herein, we present a case of a healthy young woman who developed chronic bilateral dacryoadenitis shortly after experiencing a mild SARS-CoV-2 infection.

## Case report

A 26-year-old Arab female patient presented to Infectious diseases clinic with bilateral upper lid swelling, erythema, and ptosis of 10-week duration. The patient had a mild SARS-CoV-2 infection 3 months before presentation, diagnosed by a positive polymerase chain reaction (PCR) test. Her symptoms of Covid-19 were mainly mild cough and fever. The patient noted painless swelling on the left eyelid approximately 2 weeks after the Covid-19 diagnosis, which gradually progressed into both lids. She was initially treated with antibiotics without a clear benefit and was later given steroids for a week, on which she reported a partial response. Upon presentation, she denied fever or chills during her illness period, and her systemic review of symptoms was unremarkable. The patient denied having any facial filler injections in the past.

**Fig. 1:**
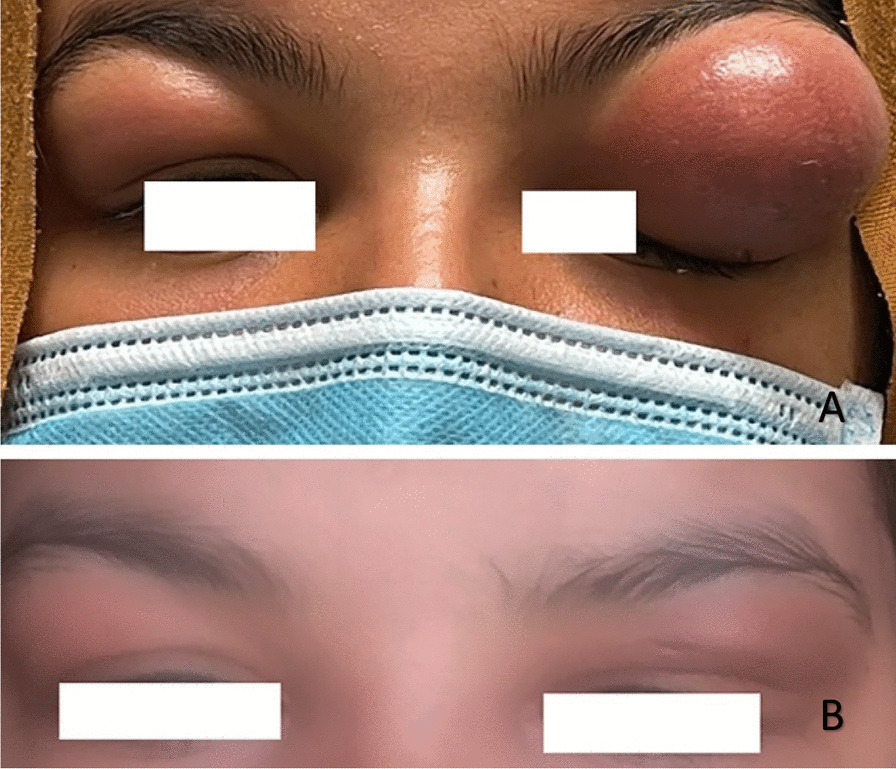
**A** Bilateral eyelid swelling; more aggressive on the left side. B Marked resolution in both swelling after 2 weeks of steroid therapy

The patient's physical examination showed bilateral eyelid oedema, erythema, tenderness, and ptosis, with more severe symptoms on the left side (Fig. 1A). She had a decreased visual acuity on the left side without limitations in both eyes' ocular muscle movements. Her vital signs, including temperature, blood pressure, and respiratory rate, were normal.

**Fig. 2:**
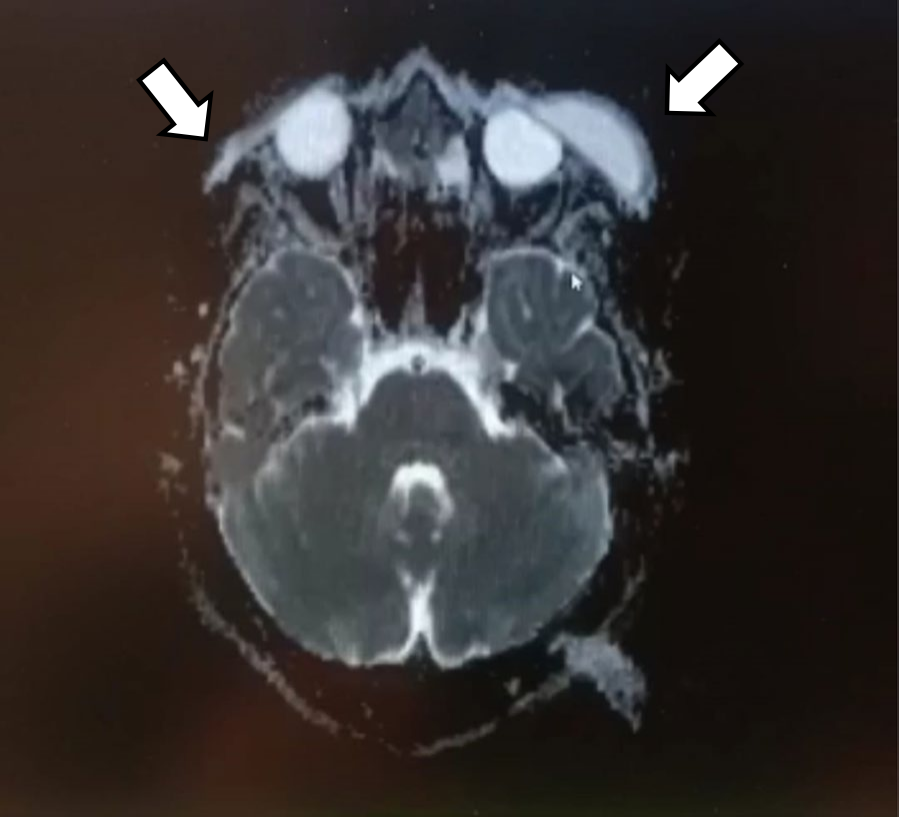
Magnetic resonance imaging brain T2 image view showing enlargement if the bilateral lacrimal glands (arrows), with a left sided collection at the site left lacrimal gland (left-sided arrow)

The results of the MRI orbit with gadolinium showed that the patient had inflammation in both of their lacrimal glands, with a collection on the left lacrimal gland measuring 3 × 2 × 2 cm (Fig. 2). The patient's laboratory work-up showed a normal white blood cell count of 10,500/mm^3^, with 61% neutrophils and 29% lymphocytes. Additionally, the electrolytes, liver function tests, bilirubin, and creatinine level were all within normal ranges. However, her C-RP and ESR levels were elevated, measuring at 4.6 mg/dl and 107 mm/hour, respectively. Auto-immune workup including ANA profile, Anti-dsDNA, ACE, C-ANCA, and P-ANCA was negative. Surgical drainage was performed to relieve pain and collect samples for testing. The samples were sent for microbiology and histopathology testing. After the surgery, she was discharged on oral clindamycin 450 mg PO every 8 hours pending microbiology results that came positive for *Staphylococcus hominis.* The histopathology report showed that there was an increase in small, capillary-sized vessels with an abundance of chronic inflammatory cells, mainly lymphocytes, plasma cells, and eosinophils. The pathology was negative for malignancy or granulomatous process. Based on the patient's chronic bilateral pathology, lack of systemic symptoms, and normal white blood count, it was determined that she had an inflammatory condition with a superimposed bacterial infection. For that, she was started on prednisone of 1 mg per kg per day orally for 2 weeks with a tapered dose to be decided on a clinical basis. The oral clindamycin was stopped after seven days. The patient exhibited progressive improvement during the follow-up visits after two and four weeks of starting steroids (Fig. 1 B).

## Discussion

Dacryoadenitis can occur as either an acute or chronic condition affecting one or both lacrimal glands [3]. Acute dacryoadenitis is caused primarily by viral illnesses like Epstein-Barr virus, Cytomegalovirus, Mumps or Varicella infection. It is a self-limited infection that usually affects the bilateral lacrimal gland [3]. The inflammation resolved spontaneously within two to four weeks [3]. Bacterial dacryoadenitis is a suppurative infection that causes a unilateral tender eyelid swelling with or without purulent discharge. The causative microorganisms are primarily *Staphylococcus aureus*, Staphylococcus coagulase negative and *Streptococcus pneumonia* [3, 4].Other microorganisms include gram-negative bacilli, *Haemophilus influenzae*, and *Moraxella catarrhalis* [3, 4]. Back to our patient, the chronicity of illness plus the bilateral involvement of lacrimal glands points against a bacterial infection. The positive culture of Staphylococcus epidermidis can be either a contaminant or a superimposed bacterial infection; however, this cannot be the sole pathology. Besides, the gradual improvement in her eyelid edema continued over the four-week follow-up where she was on steroids only.

In contrast to acute dacryoadenitis, chronic dacryoadenitis is caused primarily by auto-immune diseases, including Sjogren’s disease, Sarcoidosis, or IgG4-related auto-immune disorder [5]. Inflammatory dacryoadenitis presents with painless lacrimal gland swelling, which typically affects both sides [3, 5]. The patients will also presents with systemic signs and symptoms related to the primary disease [3].If there is suspicion of autoimmune dacryoadenitis, it's recommended to conduct an autoimmune work-up that includes testing for ANA profile, C-ANCA, P-ANCA, and ACE. If the workup did not reveal a diagnosis, a lacrimal gland biopsy should be considered. The management of inflammatory dacryoadenitis is steroids. In refractory cases, other treatment options including methotrexate, radiotherapy, or rituximab may help. The clinical differences and treatment options for viral, bacterial and inflammatory dacryoadenitis are summarized below (Table 1). In our patient, she denied any systemic signs or symptoms that suggested auto-immune disease with a lacrimal gland involvement. In addition to that, the auto-immune workup was negative and the histopathology report did not show specific findings like granuloma to suggest sarcoidosis or granulomatosis with polyangiitis. Finally, our patient’s tissue pathology was negative for malignancy.

**Table 1:** Summary of the clinical differences and treatment options for viral, bacterial and inflammatory dacryoadenitis

	Viral dacryoadenitis	Bacterial dacryoadenitis	Inflammatory dacryoadenitis
Onset	Acute	Acute	Chronic
Age	Children-young adults	Adults	Adults
Laterality	Bilateral or unilateral	Unilateral	Bilateral
Clinical picture	Fever plus tender eyelid swelling with eye discharge and conjunctivitis	Fever plus tender swelling with purulent discharge	Non-tender bilateral swelling plus systemic signs and symptoms related to the primary disease
Management	Supportive therapy	Antibiotic therapy with anti-staphylococcal coverage plus drainage in case of abscess formation	Steroids In refractory cases, consider methotrexate, radiotherapy or rituximab

Dacryoadenitis was linked to SARS-CoV-2 infection in several case reports [6–9]. Diaz *et al.* documented a case of a 22-year-old man who presented with acute dacryoadenitis and a positive SARS-CoV-2 IgG after a recent history of exposure to the virus [6]. Similarly, Demarkarian *et al.* reported a case of a 6-month-old child who had bilateral lacrimal gland inflammation associated with acute SARS-CoV-2 infection [8]. Kase *et al.* proved in their retrospective case–control the overexpression of the ACE 2 receptors in the lacrimal gland, which makes it a potential target for SARS-CoV-2 infection [9]. In addition, the immunohistochemistry study demonstrated immunoreactivity for SARS-CoV-2 nucleocapsid protein in the stromal cells of the patient with COVID-19-related chronic dacryoadenitis even after 6 months of the infection [9]. Based on these findings, they concluded that dacryoadenitis might be part of post-Covid-19 infection. Their conclusion is also supported by the reports demonstrating the occurrence of dacryoadenitis after SARS-CoV-2 vaccination [10, 11]. The recommended management is steroids, but the patient's clinical response should guide the duration of treatment [6, 8, 9].

## Conclusion

To sum up, chronic dacryoadenitis can be caused by recent exposure to SARS-CoV-2, whether through infection or vaccination. It's considered part of the inflammatory process after COVID-19 infection. The treatment is steroid therapy; however, the duration of therapy ought to be based on the patient's response.

## Data Availability

Not applicable.

## Abbreviations

SARS-CoV-2: Severe acute respiratory syndrome coronavirus 2
PCR: Polymerase chain reaction
ANA: Antinuclear antibody
ACE: Angiotensin converting enzyme
C-ANCA: C-antineutrophil cytoplasmic antibodies
P-ANCA: Perinuclear anti-neutrophil cytoplasmic antibodies
MRI: Magnetic resonance imaging
